# Characterization of HPV Vaccination Uptake by Birth Cohorts in a Healthcare System in Pennsylvania: A Post-COVID-19 Pandemic Historical Cohort Assessment

**DOI:** 10.1177/10732748251359401

**Published:** 2025-07-19

**Authors:** Josheili Y. Llavona-Ortiz, Benjamin Fogel, Lauren J. Van Scoy, Casey N. Pinto, Wen-Jan Tuan, Sandeep Pradhan, William A. Calo

**Affiliations:** 1 Department of Public Health Sciences, 12310 Penn State College of Medicine, Hershey, PA, USA; 2 University of Arizona Cancer Center, Tucson, AZ, USA; 3 Department of Pediatrics, 571415 Penn State College of Medicine, Hershey, PA, USA; 4 Departments of Medicine and Humanities, 12310 Penn State College of Medicine, Hershey, PA, USA; 5 Department of Family and Community Medicine, 12310 Penn State College of Medicine, Hershey, PA, USA; 6 Penn State Cancer Institute, Hershey, PA, USA

**Keywords:** cancer prevention, childhood vaccines, COVID-19 pandemic, HPV vaccine, vaccination trends

## Abstract

**Background:**

COVID-19 pandemic-related disruptions in primary care caused delays in in-person well visits for patients that were age-eligible to receive recommended preventive healthcare services. Vaccine schedules impacted by disruptions led to delays in administration of human papillomavirus (HPV), tetanus, diphtheria, and acellular pertussis (Tdap), and quadrivalent meningococcal (MenACWY) vaccines, but some studies report inconsistent findings. Our study sought to characterize changes in HPV vaccination patterns during the COVID-19 pandemic by birth cohorts in South Central Pennsylvania, compared to changes in Tdap and MenACWY vaccination.

**Methods:**

Our methodological approach consisted of survival analysis techniques, such as Kaplan-Meier survival curves and Cox proportional hazards models. Patient data were analyzed by separate birth cohorts and groups of birth cohorts, with a primary interest on younger birth cohorts during the COVID-19 pandemic.

**Results:**

Demographic and vaccination data from 29 928 patients born between January 1, 2000 and December 31, 2014 were analyzed. Overall, 80.1% had been vaccinated against Tdap, 81.8% against meningitis, and 67.1% against HPV by the age of 17. The adjusted hazard ratio (aHR) for HPV vaccine initiation by age 12 was the highest for patients born between 2009 and 2010 (aHR = 2.52; 95% CI: 2.34, 2.70), when compared to patients born between 2000 and 2002. However, this observation was also accompanied by a slowed increase in uptake from 63.5% (birth cohort 2006-2008) to 65.2% (birth cohort 2009-2010).

**Conclusion:**

Understanding changes in vaccination uptake by birth cohorts informs the efforts of researchers and primary care practitioners to identify best practices tailored to the vaccination gaps for each group.

## Plain Language Summary

Our study provides information regarding HPV vaccination uptake throughout birth cohorts from 2000 to 2014 to understand changes in vaccination trends in the post-pandemic era. To compare, we also evaluated vaccination behaviors for Tdap and MenACWY vaccines in these cohorts.

## Introduction

Human papillomavirus (HPV) vaccination rates have been consistently lower than other childhood vaccines since it was initially approved in 2006.^1–3^ Between 2005 and 2006, the tetanus, diphtheria, acellular pertussis (Tdap), and the quadrivalent meningococcal (MenACWY) vaccines were recommended for children between the ages of 11 and 12 years old by the Advisory Committee on Immunization Practices (ACIP).^4^ By 2009, while Tdap and MenACWY achieved US vaccination rates of 56% and 54%, respectively, the HPV vaccine initiation (receiving at least one dose) rate was only 44%.^5^ This lower rate of HPV vaccination was still observed in 2022, with Tdap vaccine administration and MenACWY vaccine initiation rates of 90% and 89% respectively, and HPV vaccine initiation rates of only 76%.^6^

During the COVID-19 pandemic, interruptions of in-person well visits were accompanied with sharp declines in vaccination rates for all vaccines.^7–10^ A US-based study found that parents of children between 2 and 12 years old were 82% more likely to report that their child had missed a healthcare visit (adjusted prevalence ratio [aPR]: 1.82; 95% CI: 1.47-2.26). Further, more than 30% of parents said their child had missed at least one routine recommended vaccine.^10^ Additionally, national insurance claims data showed that HPV vaccination coverage decreased more than the coverages for Tdap and MenACWY vaccines during 2020.^11^ Other studies have demonstrated these declines in vaccination rates and how this impacted childhood routine vaccines differently, with reports from the Centers for Disease Control and Prevention (CDC) also describing steeper drops in national HPV vaccination rates within specific population subgroups (eg, differences by health insurance type or area of residence).^6,12^

These observations were followed by unequal recovery patterns to reach pre-pandemic levels after the height of the COVID-19 pandemic, especially for HPV vaccination.^6^ Although the CDC has published reports about the number of Tdap and MenACWY vaccine provider orders surpassing those prior to the pandemic, this has not been the case for HPV vaccine orders.^6^ Based on 2022 vaccine data from the Vaccines for Children (VFC) federal program, HPV vaccine orders were 10% less than levels achieved prior to the COVID-19 pandemic, suggesting a potential decline in vaccine administration.^13^ Additionally, comparisons across birth cohorts show that children born in 2008 observed decreases in coverage for Tdap, MenACWY, and HPV vaccination compared to those born in 2007 by ages 13 and 14.^6^ Meanwhile, pre-pandemic vaccination coverage levels for these three vaccines were achieved for children born in 2009 by age 13, compared to those born in 2007.^6^

Survival analyses have been previously used to identify changes and delays in vaccination patterns.^6,12,14,15^ Some of these assessments have been conducted using birth cohorts, as this facilitates comparisons utilizing similar time-to-event periods across participants.^6,12^ Combining these approaches, our study sought to assess HPV vaccination rates compared to other routine childhood vaccines (Tdap and MenACWY) throughout the COVID-19 pandemic. We hypothesized that HPV vaccine initiation coverage would have decreased more among younger birth cohorts compared to the vaccination coverage for Tdap and MenACWY in these same birth cohorts. Moreover, our hypotheses related to sociodemographic variables and vaccination uptake for all three vaccine behaviors assessed were that significant disparities exist between sex, race, ethnicity, and primary health insurance. Additionally, we hypothesized that the HPV vaccine initiation uptake would be confirmed to have decreased using survival analysis approaches, after adjusting for sociodemographic characteristics and stratifying by groups of birth cohorts.

## Materials and Methods

### Study Design and Population

The study period for this historical cohort assessment was January 1, 2018 to December 31, 2023. All patients turning 9 to 17 years old in any given calendar year of the defined study period and had at least one active visit within the study period were included. Since age is a demographic variable that changes across time, the study involved a dynamic cohort, with children included in the study upon turning 9 years old and dropped (or censored) upon turning 18 years old between the years of 2018 and 2023. All patients were seen at one of 17 primary care clinics affiliated with a single health system in South Central Pennsylvania. Data was obtained through electronic medical record (EMR) abstraction with patient information including demographic characteristics (date of birth, sex, primary insurance, race, and ethnicity) and vaccine administration date.

### Vaccination Definition

Historical vaccination data was obtained for Tdap, MenACWY, and HPV. The following Current Procedural Terminology (CPT) codes were used to identify vaccine orders: HPV CPT Codes – 90649, 90650, 90651; Meningococcal CPT Codes – 90734, 90620, 90619, 90621; Tdap CPT Code – 90715.^16^ The Advisory Committee on Immunization Practices (ACIP) recommendation guidelines were used to determine if the reported vaccine administration date was appropriate based on the child’s age at the time.^17^ HPV vaccine initiation was defined as receiving at least one dose between the ages of 9 and 17. Tdap administration and MenACWY vaccine initiation were defined as the report of one dose between the ages of 11 and 17. Administration dates suggesting that the child got any of these vaccines outside the recommended age were considered as missing to account for erroneous data entry or administration not following ACIP guidelines.

### Data Analysis

Sociodemographic data and vaccine uptake for all unique patients were assessed using frequencies and percentages. These data were then stratified and reported by birth year of each patient to characterize sociodemographic variables and vaccine receipt across birth year cohorts. Unadjusted Cox proportional hazards models were also performed with each sociodemographic characteristic to assess the association of each with the vaccination behaviors of interest: HPV vaccine initiation, Tdap vaccine administration, and MenACWY vaccine initiation. Consistent with vaccine recommendation guidelines, patients reported as receiving the first HPV vaccine before age 9 were recategorized as ‘missing’ (n = 65). Similarly, patients reported as receiving Tdap or Meningococcal vaccines before age 11 were recategorized as ‘missing’ (n = 3476 and n = 965, respectively).

To calculate the cumulative occurrence of vaccination by vaccine of interest (HPV, Tdap, and MenACWY), Kaplan-Meier analyses were conducted to understand overall vaccination behaviors upon children becoming eligible to be immunized.^14,15,18^ Children were grouped by each birth cohort separately and birth cohort groups based on birth year, deploying a similar approach utilized by researchers when analyzing the National Immunization Survey – Teen (NIS – Teen) data.^6,12,18,19^ Analyzing data by birth cohorts ensures patients are compared to others with the same opportunity for vaccination. Patients enter the study on their ninth birthday or 1/1/2018, whichever comes second. Patients exit the study (or are censored) the day before their 18^th^ birthday or 12/31/2023, whichever comes first. For example, a patient born on 1/1/2012 would enter the study on 1/1/2021 and exit the study on (12/31/2023). Time-to-event for each patient was defined by the number of days that passed upon first being age-eligible to get the vaccine of interest, according to ACIP guidelines, up until vaccine administration.

Kaplan-Meier curves were conducted for HPV vaccine initiation, Tdap vaccine administration, and MenACWY vaccine initiation based on age eligibility by vaccine until the day before turning 18 years old. Presenting Kaplan-Meier curves by birth cohort provides visualization of changes in vaccination behaviors across time. Cox proportional hazard models were conducted for HPV vaccine initiation, Tdap vaccine administration, and MenACWY vaccine initiation based on age eligibility by vaccine until the age of 12 by group of birth cohorts. By grouping the birth years, it is possible to simplify the interpretation of the findings and reduce the comparison groups. To verify that the proportional hazards assumptions were met for the main predictor (group of birth cohorts) and covariates of interest, log-negative-log plots of the Kaplan-Meier estimates were visually examined to verify if curves were parallel to each other across time.^20^ Schoenfeld residuals, Cox-Snell residuals, and deviance residuals were also assessed to verify that the proportional hazards assumptions were met and no major violations to the assumptions were observed. Through Cox proportional hazard models, the time-to-event occurrence in each group of birth cohorts (2000-2002, 2003-2005, 2006-2008, 2009-2010) was compared to a baseline group (reference group of birth cohorts: 2000-2002). The variables of race (White/Non-White) and insurance (Commercial/Medicaid or Uninsured) were regrouped into two categories to facilitate the interpretations of the models. All patients were assigned at time 0, the day in which they turned 9 (for HPV vaccination) or 11 (for Tdap and MenACWY vaccination). To set a similar end point for all patients in these models, vaccination behavior assessment was done between 9 and 12 years old for HPV vaccination, and between 11 and 12 years old for Tdap and MenACWY. Given that normally all children are eligible for these vaccines upon age eligibility, which allows the assumption that the baseline hazard is the same for all patients and the hazard across time is fairly constant, it is determined that Cox proportional hazard models are appropriate. The protocol for this study was approved by the Pennsylvania State University’s Institutional Review Board on September 12, 2023 (Study #00022182). All data analyses were conducted using the Statistical Analysis Software (SAS) 9.4 and SAS OnDemand for Academics.^21,22^ The reporting of this study followed STROBE guidelines.^23^

## Results

### Population Characteristics

A total of 29 928 patients were included in the analysis. The dataset included data for patients born between January 1, 2000 and December 31, 2014. Roughly half (51.3%) of patients were reported as female, 60.1% were reported as White, 10.6% as Black, 4.4% as Asian, and 24.9% as Other (eg, Native American, Pacific Islander, and Other). Most patients (89.8%) were reported as non-Hispanic and 59.4% of patients had commercial insurance. Table 1 has sociodemographic data presented by birth cohort to characterize the distribution of data for each sub-group.

**Table 1.** Patient Demographics by Birth Cohort (Defined by Year of Birth) – *N* = 29 928

Birth year	2000	2001	2002	2003	2004	2005	2006	2007
Total patients (n)	2178	2244	2166	2099	2111	2142	2159	2146
Demographic variable	*n* (%)	*n* (%)	*n* (%)	*n* (%)	*n* (%)	*n* (%)	*n* (%)	*n* (%)
Sex								
Male	981 (45.0)	1062 (47.3)	1085 (50.1)	1021 (48.6)	1027 (48.7)	1071 (50.0)	1077 (49.9)	1055 (49.2)
Female	1197 (55.0)	1182 (52.7)	1081 (49.9)	1078 (51.4)	1084 (51.3)	1071 (50.0)	1082 (50.1)	1091 (50.8)
Race								
White	1466 (67.3)	1484 (66.1)	1368 (63.2)	1338 (63.7)	1327 (62.9)	1298 (60.6)	1291 (59.8)	1267 (59.0)
Black	198 (9.1)	223 (9.9)	214 (9.9)	207 (9.9)	201 (9.5)	245 (11.4)	235 (10.9)	260 (12.1)
Asian	73 (3.3)	69 (3.1)	77 (3.5)	70 (3.3)	76 (3.6)	80 (3.7)	83 (3.8)	84 (3.9)
Other	441 (20.3)	468 (20.9)	507 (23.4)	484 (23.1)	507 (24.0)	519 (24.2)	550 (25.5)	535 (24.9)
Ethnicity								
Hispanic	200 (9.2)	187 (8.3)	196 (9.1)	195 (9.3)	191 (9.1)	194 (9.1)	252 (11.7)	248 (11.6)
Non-Hispanic	1978 (90.8)	2057 (91.7)	1970 (90.9)	1904 (90.7)	1920 (90.9)	1948 (90.9)	1907 (88.3)	1898 (88.4)
Insurance								
Commercial	1406 (64.5)	1485 (66.2)	1379 (63.7)	1352 (64.4)	1279 (60.6)	1256 (58.6)	1253 (58.0)	1252 (58.3)
Medicaid	459 (21.1)	523 (23.3)	577 (26.6)	545 (26.0)	617 (29.2)	705 (32.9)	789 (36.5)	813 (37.9)
Uninsured	313 (14.4)	236 (10.5)	210 (9.7)	202 (9.6)	215 (10.2)	181 (8.5)	117 (5.4)	81 (3.8)
Birth year	2008	2009	2010	2011	2012	2013	2014	

Total patients (*n*)	2010	1949	1907	1898	1779	1575	1565	
Demographic variable	*n* (%)	*n* (%)	*n* (%)	*n* (%)	*n* (%)	*n* (%)	*n* (%)	
Sex								
Male	1045 (52.0)	1008 (51.7)	953 (50.0)	959 (50.5)	906 (50.9)	812 (51.6)	806 (51.5)	
Female	965 (48.0)	941 (48.3)	954 (50.0)	939 (49.5)	873 (49.1)	763 (48.4)	759 (48.5)	
Race								
White	1206 (60.0)	1133 (58.1)	1073 (56.3)	1072 (56.5)	964 (54.2)	863 (54.8)	843 (53.9)	
Black	230 (11.4)	224 (11.5)	220 (11.5)	223 (11.8)	186 (10.5)	161 (10.2)	139 (8.9)	
Asian	81 (4.0)	91 (4.7)	94 (4.9)	99 (5.2)	117 (6.6)	97 (6.2)	121 (7.7)	
Other	493 (24.5)	501 (25.7)	520 (27.3)	504 (26.5)	512 (28.8)	454 (28.8)	462 (29.5)	
Ethnicity								
Hispanic	243 (12.1)	223 (11.4)	192 (10.1)	199 (10.5)	194 (10.9)	149 (9.5)	182 (11.6)	
Non-Hispanic	1767 (87.9)	1726 (88.6)	1715 (89.9)	1699 (89.5)	1585 (89.1)	1426 (90.5)	1383 (88.4)	
Insurance								
Commercial	1160 (57.7)	1117 (57.3)	1045 (54.8)	1054 (55.5)	947 (53.2)	895 (56.8)	896 (57.3)	
Medicaid	779 (38.8)	767 (39.4)	808 (42.4)	791 (41.7)	788 (44.3)	636 (40.4)	633 (40.4)	
Uninsured	71 (3.5)	65 (3.3)	54 (2.8)	53 (2.8)	44 (2.5)	44 (2.8)	36 (2.3)	

### Vaccine Uptake

Across the entire study population, 67.1% initiated the HPV vaccination series, 80.1% received the Tdap vaccine, and 81.8% initiated their MenACWY vaccination series. Table 2 has vaccination data presented by birth cohort to characterize the changes in uptake by year. Unadjusted Cox proportional hazards models were used to assess associations between sociodemographic variables and vaccination against HPV, Tdap, and meningococcal diseases. Statistically significant differences (all with *P* < 0.01) were observed for all sociodemographic variables across vaccines except for patient’s sex related to Tdap and MenACWY vaccines. For all categories of race, ethnicity, and insurance, Tdap and MenACWY vaccine administration occurred less among Non-Whites (race), Hispanics (ethnicity), and publicly insured or uninsured patients (insurance). When assessing the same sociodemographic associations with HPV vaccine initiation, vaccination occurred significantly less among males while it occurred significantly more among Non-Whites (race), Hispanics (ethnicity), and publicly insured or uninsured patients (insurance). These data are presented in Appendix A.

**Table 2.** Vaccine Uptake for HPV, Tdap, and MenACWY Vaccines by Birth Year

Birth year	2000	2001	2002	2003	2004	2005	2006	2007
Vaccine	*n* (%)	*n* (%)	*n* (%)	*n* (%)	*n* (%)	*n* (%)	*n* (%)	*n* (%)
HPV								
Initiation (≥1)	1434 (66.1)	1524 (68.2)	1550 (71.7)	1525 (73.0)	1610 (76.5)	1630 (76.2)	1712 (79.5)	1686 (78.8)
Tdap								
One dose	1660 (83.8)	1656 (84.5)	1620 (88.8)	1595 (89.7)	1617 (93.6)	1646 (94.1)	1683 (95.9)	1659 (94.8)
Meningococcal								
Initiation (≥1)	1823 (86.6)	1893 (87.2)	1897 (90.7)	1872 (92.9)	1935 (94.4)	1958 (95.0)	1984 (96.9)	2003 (96.2)

Birth year	2008	2009	2010	2011	2012	2013	2014	
Vaccine	*n* (%)	*n* (%)	*n* (%)	*n* (%)	*n* (%)	*n* (%)	*n* (%)	
HPV								
Initiation (≥1)	1534 (76.4)	1471 (75.7)	1346 (70.7)	1255 (66.2)	967 (54.4)	530 (33.7)	342 (21.9)	
Tdap								
One dose	1588 (94.2)	1692 (92.9)	1649 (90.5)	1609 (87.9)	1258 (73.1)	—	—	
Meningococcal								
Initiation (≥1)	1827 (93.3)	1738 (92.0)	1657 (89.8)	1598 (86.4)	1259 (72.6)	—	—	

Note: HPV vaccination data, *n* = 29 863; Tdap vaccination data, n = 26 452; Meningococcal vaccination data, *n* = 28 966; The use of ‘—’ in the table reflects birth cohorts that were not age eligible for Tdap and MenACWY vaccines (≥11 years old) per ACIP guidelines during the study period.

### Survival Analysis

**Figure 1.**
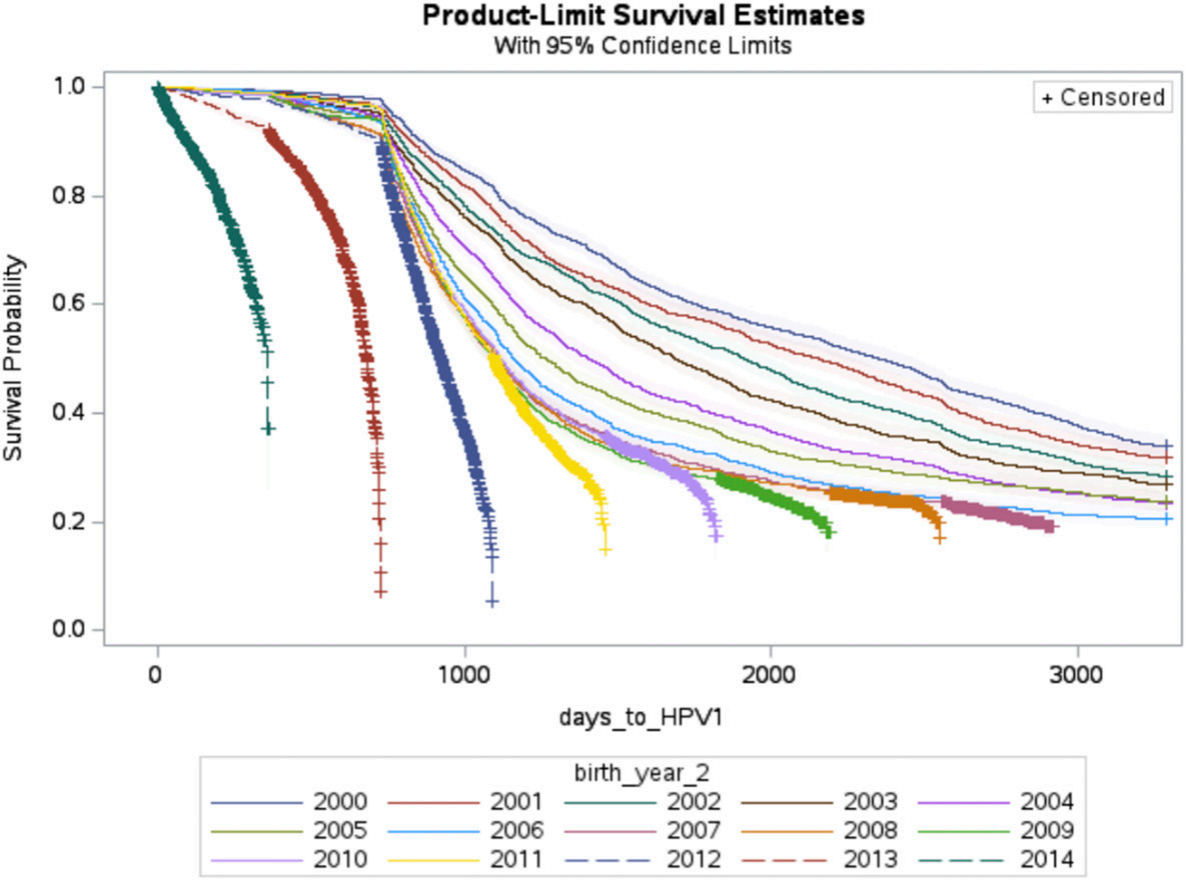
Kaplan-Meier Curves of HPV Vaccine Initiation by Birth Year

Kaplan-Meier curves for HPV vaccine initiation across birth cohorts from 2000 to 2014 are shown in Figure 1. Survival curves for Tdap and MenACWY vaccination across all birth cohorts have been included for comparison in Appendixes B and C. For birth cohorts 2000 to 2006, all children made it at least to the first day of being 17 years of age. Survival probability, in this case referring to the probability of not getting vaccinated, decreased across all birth cohorts compared to the reference birth year (2000). About 56% of patients born in 2000 got vaccinated against HPV by the age of 17, compared to almost 80% of patients born in 2006. Patients from birth cohorts 2007 through 2011 were between the ages of 12 and 16 by 2023, therefore having less time to provide vaccination data compared to previous cohorts. Within these birth cohorts, between 56% and 79% of patients had at least initiated the HPV vaccination series. Lastly, patients from birth cohorts 2012 to 2014 were between the ages of 9 and 11 by 2023, thus joining the study time between 2021 and 2023. While patients from birth cohort 2012 followed vaccination trends from previous birth cohorts, subsets of patients from birth cohorts 2013 and 2014 started vaccinating earlier (between the ages of 9 and 10). Inclusion of cohorts with less complete data (ie, children born in 2007 or later) allowed assessment of preliminary trends of vaccination compared to birth cohorts 2006 and earlier.

The Cox Proportional Hazards models, with vaccine administration for each vaccine as outcome variable and groups of birth cohorts as primary predictor, were adjusted for all sociodemographic variables included in the study (Table 3). Appropriate use of these models was assessed through model diagnostics to assess the proportional hazards assumptions for all variables and determining that all log-negative-log plots with Kaplan-Meier estimates were identified as being parallel across time (Appendixes G-I). Based on the overall assessment of the model diagnostics, it was determined that no major violations to proportional hazards assumptions were identified. These models sought to assess the instantaneous risk (or hazard) of receiving each of the vaccines of interest by the day before turning 13 years old. Compared to the group of birth cohorts of 2000-2002, all subsequent groups of birth cohorts had higher hazards of initiating HPV vaccination at any point before or at age 12. Compared to patients born between 2000 and 2002, the hazard ratio to initiation HPV vaccination by age 12 was the highest for patients born between 2009 and 2010 (aHR = 2.52; 95% CI: 2.34, 2.70). On the other hand, the hazard ratios for Tdap vaccine administration at any point at age 12 consistently increased for the subgroups of patients born between 2003 and 2005 (aHR = 1.33; 95% CI: 1.27, 1.40) and 2006 to 2008 (aHR = 1.58; 95% CI: 1.50, 1.66), followed by a decrease in the subgroup of patients born between 2009 and 2010 (aHR = 1.41; 95% CI: 1.33, 1.49). A similar increase-to-decrease observation was found for MenACWY vaccine administration across these birth cohorts. Additional Kaplan-Meier survival curves modeling data for birth cohorts from 2000 to 2010 for HPV vaccine initiation between 9 and 12 years old, and Tdap and MenACWY vaccine administration between 11 and 12 years old are included in Appendix D through F.

**Table 3.** Cox Proportional Hazards Models for Tdap, MenACWY, and HPV Vaccination by 12 Years Old

Birth year group	Tdap vaccine administration			Meningococcal vaccine initiation			HPV vaccine initiation		
	Overall significance of model: <0.0001			Overall significance of model: <0.0001			Overall significance of model: <0.0001		
	*n*/*N* (%)	aHR	(95% CI)	*n*/*N* (%)	aHR	(95% CI)	*n*/*N* (%)	aHR	(95% CI)
2000-2002	4395/5765 (76.2)	Ref	—	4847/6367 (76.1)	Ref	—	2317/6567 (35.3)	Ref	—
2003-2005	4507/5255 (85.8)	1.33	(1.27, 1.40)	5173/6127 (84.4)	1.27	(1.22, 1.33)	3172/6330 (50.1)	1.60	(1.50, 1.71)
2006-2008	4674/5190 (90.1)	1.58	(1.50, 1.66)	5417/6088 (89.0)	1.53	(1.46, 1.60)	4003/6304 (63.5)	2.47	(2.32, 2.63)
2009-2010	3219/3644 (88.3)	1.41	(1.33, 1.49)	3271/3735 (87.6)	1.44	(1.46, 1.52)	2508/3844 (65.2)	2.52	(2.34, 2.70)

Note: All models were adjusted for child’s sex, race, ethnicity, and insurance. ACIP vaccine administration guidelines were used to define vaccine administration practices. HPV vaccine initiation was assessed between 9 and 12 years old. Tdap/MenACWY vaccine administration was assessed between 11 and 12 years old.

aHR, adjusted hazard ratio; CI, confidence interval.

## Discussion

Since the pandemic, calls for more research to characterize the impact of the COVID-19 pandemic on routine vaccinations have been put forward to understand changes in vaccination uptake.^6,12^ In this study, we focused on characterizing vaccination behaviors by birth cohorts descriptively and using survival analysis approaches. Through descriptive statistics, the consistent lag of HPV vaccination compared to Tdap and MenACWY vaccination across birth cohorts was confirmed. There is a consistent difference in coverage, overall ranging between 15% and 20%, when comparing HPV vaccination to Tdap and MenACWY vaccination (Table 2). Using Kaplan-Meier curves, visualization of differences in routine childhood vaccination patterns have been provided among cohorts of patients between 9 and 17 years old between 2018 and 2023. As seen throughout the literature, HPV vaccination uptake behaviors (Figure 1) are different than vaccination behaviors for Tdap (Appendix B) and MenACWY (Appendix C). While behaviors for Tdap and MenACWY vaccination are similar and occur mainly between ages 11 and 12, behaviors for HPV vaccine initiation occur across a longer time span up until age 17. Even then, vaccination coverage against HPV by age 17 is similar to the vaccination coverage achieved against both Tdap and MenACWY by age 12. Previous HPV vaccine recommendation guidelines strongly encouraged a bundled recommendation approach, suggesting providers to recommend Tdap, HPV, and MenACWY vaccines between ages 11 and 12.^3^ When recommending multiple vaccines at once, it was not uncommon for providers to document that parents would push back on the HPV vaccine because it was not required for school entry or due to concerns of administering numerous shots.^24^

Recent findings suggest that Tdap and MenACWY vaccination uptake patterns have not significantly shifted.^6,18^ Compared to the group of birth cohorts from 2000 to 2002, aHRs for Tdap and MenACWY vaccination improved for groups of birth cohorts from 2003 to 2005 and 2006 to 2008. However, the aHR for the group of birth cohorts from 2009 to 2010 shows a decrease in uptake, suggesting a slow-down in the administration process for both Tdap and MenACWY vaccines by age 12. This finding aligns with both qualitative and quantitative findings across the US that have suggested increases in vaccine hesitancy to vaccines in general since the COVID-19 pandemic.^25,26^ Moreover, Tdap and MenACWY were added as school-required vaccines in PA in 2011 and the mandate gave parents up to 8 months to provide proof of vaccination or exemptions.^27–29^ This mandate was followed by an adjustment in 2017, which gave parents no more than 5 days to provide documentation of vaccine receipt or exemption.^27^ Increases in Tdap and MenACWY vaccination coverage, prior to the decrease observed for patients born between 2009 and 2010, may be explained by these policy changes.

Compared to Tdap and MenACWY vaccination coverage, HPV vaccination uptake continues to lag and seems to have not recovered to pre-pandemic levels. In this study, aHRs for HPV vaccine initiation by age 12 consistently increased across groups of birth cohorts, compared to the group of patients born between 2000 and 2002. Similar to vaccination patterns for Tdap and MenACWY across groups of birth cohorts, although no decrease was observed, HPV vaccine initiation uptake noticeably slowed down from the group of birth cohorts 2006 to 2008 to the group of birth cohorts 2009 to 2010. Overall findings from nationwide assessments suggest that the pandemic impact on HPV vaccination uptake, in general, has been similar to other vaccines and that uptake has not significantly changed nor consistently decreased, compared to pre-pandemic levels.^6,12,18^ For instance, the CDC’s TeenVaxView Interactive! Platform reports that trends for HPV vaccination among adolescents 13 to 17 years old decreased from 78.4% in 2020 to 76.4% in 2022 across Pennsylvania.^30^ On the other hand, data analyses of commercially insured patients show that Tdap and MenACWY vaccination rates had remained stable compared to pre-pandemic levels while HPV vaccination coverage saw an initial 6% increase by October 2020, followed by a 10.9% decrease by January 2021.^31^ Policy changes (eg, school entry requirements) for Tdap and MenACWY vaccines may have played a role for coverage to not be significantly impacted by the pandemic. This could suggest that school entry requirement policies could be beneficial for HPV vaccination to (1) increase uptake and (2) stabilize coverage in the light of nationwide disruptions.

Another potential solution to the lag in HPV vaccine initiation by age 12, compared to Tdap and MenACWY, has been to start recommending the HPV vaccine as early as 9 years old.^3,32,33^ In 2020, the American Cancer Society (ACS) updated their guidelines to encourage providers to start recommending the HPV vaccine by age 9, following the recommendation of the American Academy of Pediatrics (AAP) to do the same.^34,35^ This approach helps alleviate parental concerns of children receiving multiple vaccines at the same time and allows more time for providers to discuss parental questions about vaccines.^36,37^ Preliminary observations from our Kaplan-Meier curves (Figure 1) suggest that more providers have started to recommend the HPV vaccine between ages 9 and 10 among the younger birth cohorts (2013-2014). This finding aligns with observations by CDC indicating that younger birth cohorts require particular focus to understand vaccination behaviors post-pandemic.^6^ It is essential to conduct follow-up research on these younger birth cohorts that turned 9 and 10 years old after the height of the pandemic to characterize vaccination patterns and compare their HPV vaccination uptake behaviors to pre-pandemic cohorts.

## Strengths and Limitations

The present study was based on a robust sample size with provider-verified data with confirmed CPT codes, which allowed to conduct a comprehensive analysis of vaccination trends across 15 unique birth cohorts. Additionally, the demographic breakdown of the study population is similar to South Central Pennsylvania’s overall demography, which strengthens its representativity of the region. Moreover, the sociodemographic variables included in this study have been previously associated with vaccination uptake. By selecting the group of patients born between 2000 and 2002 as the reference group, it is possible to understand changes of vaccination across groups of birth cohorts impacted by different national policies (eg, vaccine-related school-entry policies) and events (eg, COVID-19 pandemic). In terms of limitations, for the analysis, children that were vaccinated outside the age of recommendation following ACIP guidelines were labelled as missing. This approach might have led to an underestimation of children vaccinated within the age of interest. However, findings of sensitivity analyses conducted (not shown) including children receiving vaccines within 365 days prior to ACIP age recommendation did not show differences in results. Another limitation is the lack of calculation for the study sample, although this occurred given the fact that all patients within the study period and related study sites were included. It is also important to acknowledge the potential occurrence of data entry errors which may have left out children that had received the vaccines of interest at other sites but were not reported. Nonetheless, given that these are vaccines routinely administered at children’s primary care provider offices, this represents a considerably small number of patients.^38^ Additionally, the presented data is not generalizable to the entire state as it only relates to a single healthcare system in South Central Pennsylvania. There are distinct disparities and racial/ethnic compositions across different counties in Pennsylvania that limit the generalizability of the data. Moreover, key covariates and potential confounders such as receiving a provider recommendation, vaccine hesitancy, or parents’ education were not considered in the models as these data were not available in EMR.^39^ Although no qualitative data was presented to further explain the present study findings, our research team simultaneously conducted a qualitative study regarding pandemic-related changes in HPV vaccination uptake.^40^ Twenty-five primary care team members (eg, physicians, nurses, physician assistants) from Pennsylvania were interviewed related to changes in adolescent vaccination throughout the COVID-19 pandemic, with a focus on HPV vaccination. Findings of the study provide insight into challenges faced by primary care team members when having HPV vaccine conversations with parents during the COVID-19 pandemic.

## Conclusion

Using survival analysis approaches to visualize and characterize vaccination behaviors as time-to-event outcomes is insightful to identify patterns across large periods. Such findings help inform and tailor evidence-based interventions and recommendation guidelines for vaccines and vaccine conversations. By comparing HPV vaccination behaviors to Tdap and MenACWY vaccination uptake, it is possible to characterize the uptake of the most common recommendation guidelines (eg, recommending all three vaccines together) and potential areas for improvement. For HPV vaccination, it would be imperative to follow-up with birth cohorts 2012 through 2014 to assess changes in vaccination uptake once these children turn 12 years old. Doing so would provide insight on best practices and lessons learned in these first birth cohorts under the updated guidelines for HPV vaccination. Previous and recent research efforts have been conducted to understand how starting the conversation about the HPV vaccine earlier, with parents of 9-year-old children, may provide better initiation and completion rates for HPV vaccination.^32,41^ Additional work, including qualitative interviews, should seek to understand how these potential changes in uptake would help inform educational interventions for both providers and parents.

## Supplemental Material

Supplemental Material - Characterization of HPV Vaccination Uptake by Birth Cohorts in a Healthcare System in Pennsylvania: A Post-COVID-19 Pandemic Historical Cohort Assessment Supplemental Material for Characterization of HPV Vaccination Uptake by Birth Cohorts in a Healthcare System in Pennsylvania: A Post-COVID-19 Pandemic Historical Cohort Assessment by Josheili Y. Llavona-Ortiz, Benjamin Fogel, Lauren J. Van Scoy, Casey Pinto, Wen-Jan Tuan, Sandeep Pradhan, and William A. Calo in Cancer Control
