# Supplementary material for: Characterization of HPV Vaccination Uptake by Birth Cohorts in a Healthcare System in Pennsylvania: A Post-COVID-19 Pandemic Historical Cohort Assessment
Source: Cancer Control. 2025 Jul 19;32:10732748251359401. doi: 10.1177/10732748251359401 (PMC12276464; doi:10.1177/10732748251359401)
Supplement: Supplemental Material - Characterization of HPV Vaccination Uptake by Birth Cohorts in a Healthcare System in Pennsylvania: A Post-COVID-19 Pandemic Historical Cohort Assessment [file sj-pdf-1-ccx-10.1177_10732748251359401.pdf]

Appendix A: Bivariate Analysis using Unadjusted Cox Proportional Hazards Models to Assess Association Between Sociodemographic Variables and Vaccine Behavior (9-17 years old)

	**Tdap Vaccine Administration**			**Meningococcal Vaccine Initiation**			**HPV Vaccine Initiation**		
	Total vaccinated/Total: 21,303/26,575			Total vaccinated/Total: 23,808/29,085			Total vaccinated/Total: 20,132/29,968		
	**HR**	**(95% CI)**	**p-value**	**HR**	**(95% CI)**	**p-value**	**HR**	**(95% CI)**	**p-value**
**Sex**									
Female	Ref	-	0.5065	Ref	-	0.3267	Ref	-	<0.0001
Male	1.01	(0.99, 1.04)		1.01	(0.98, 1.04)		0.92	(0.90, 0.95)	
**Race**									
White	Ref	-	<0.0001	Ref	-	<0.0001	Ref	-	<0.0001
Non-White	0.81	(0.79, 0.83)		0.82	(0.80, 0.84)		1.22	(1.19, 1.26)	
**Ethnicity**									
Non-Hispanic	Ref	-	<0.0001	Ref	-	<0.0001	Ref	-	<0.0001
Hispanic	0.87	(0.83, 0.91)		0.89	(0.85, 0.93)		1.22	(1.17, 1.28)	
**Insurance**									
Commercial	Ref	-	<0.0001	Ref	-	<0.0001	Ref	-	<0.0001
Medicaid or Uninsured	0.80	(0.78, 0.82)		0.82	(0.80, 0.84)		1.07	(1.04, 1.10)	

Denominators vary by vaccine due to adjustments for data accuracy and consistency. Patients reported as receiving the first HPV vaccine before age 9 were recategorized as ‘missing’. Patients reported as receiving Tdap or Meningococcal vaccines before age 11 were recategorized as ‘missing’.

Appendix B: Kaplan-Meier curves – Tdap Vaccine Administration (ages 11-17)

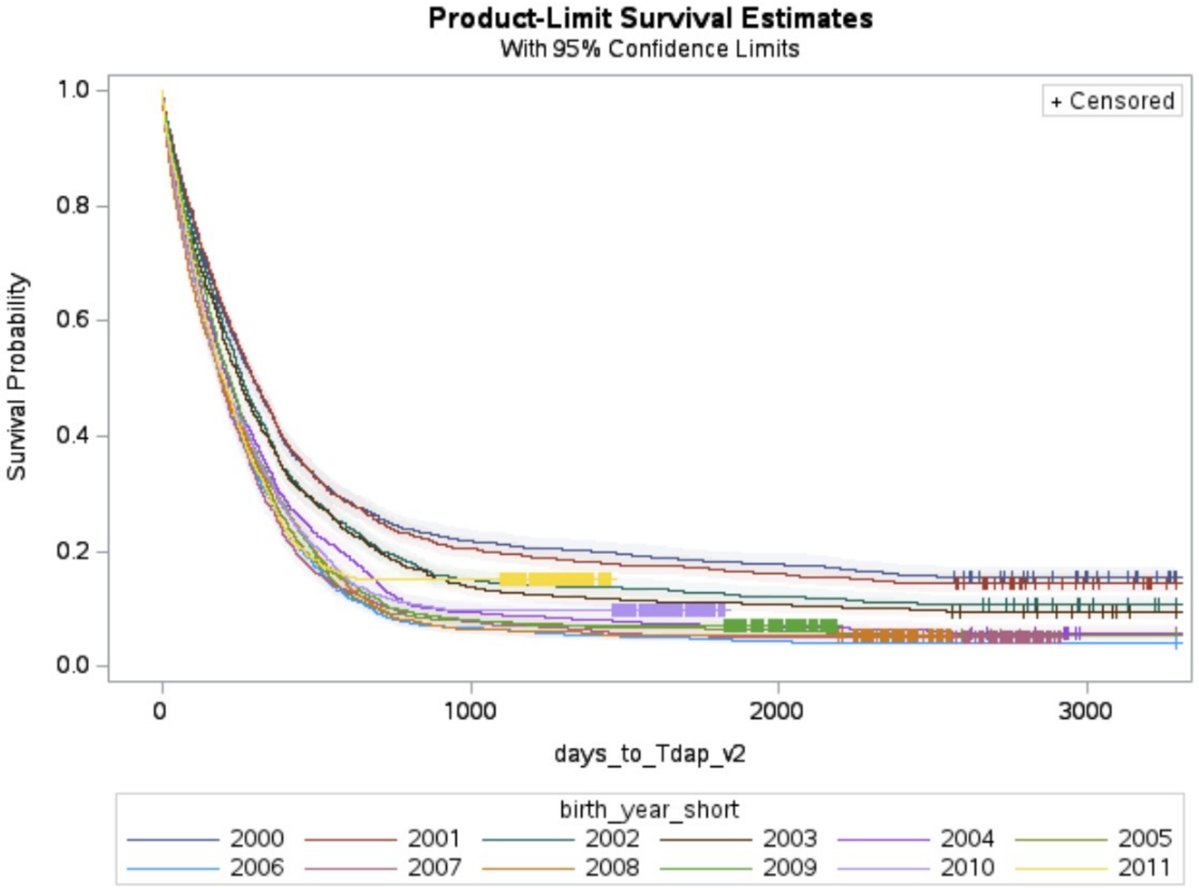

Appendix C: Kaplan-Meier curves – MenACWY Vaccine Initiation (ages 11-17)

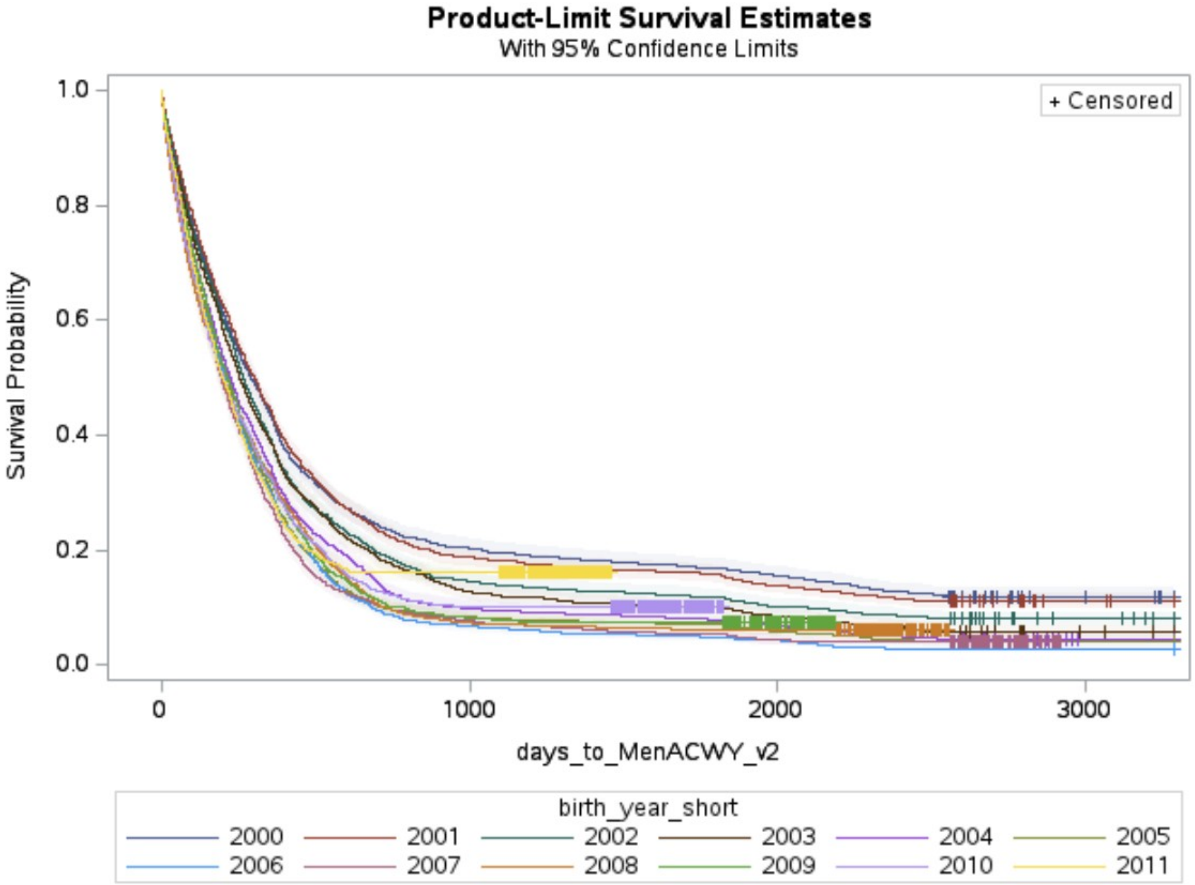

Appendix D: Kaplan-Meier curves – HPV Vaccine Initiation (ages 9-12)

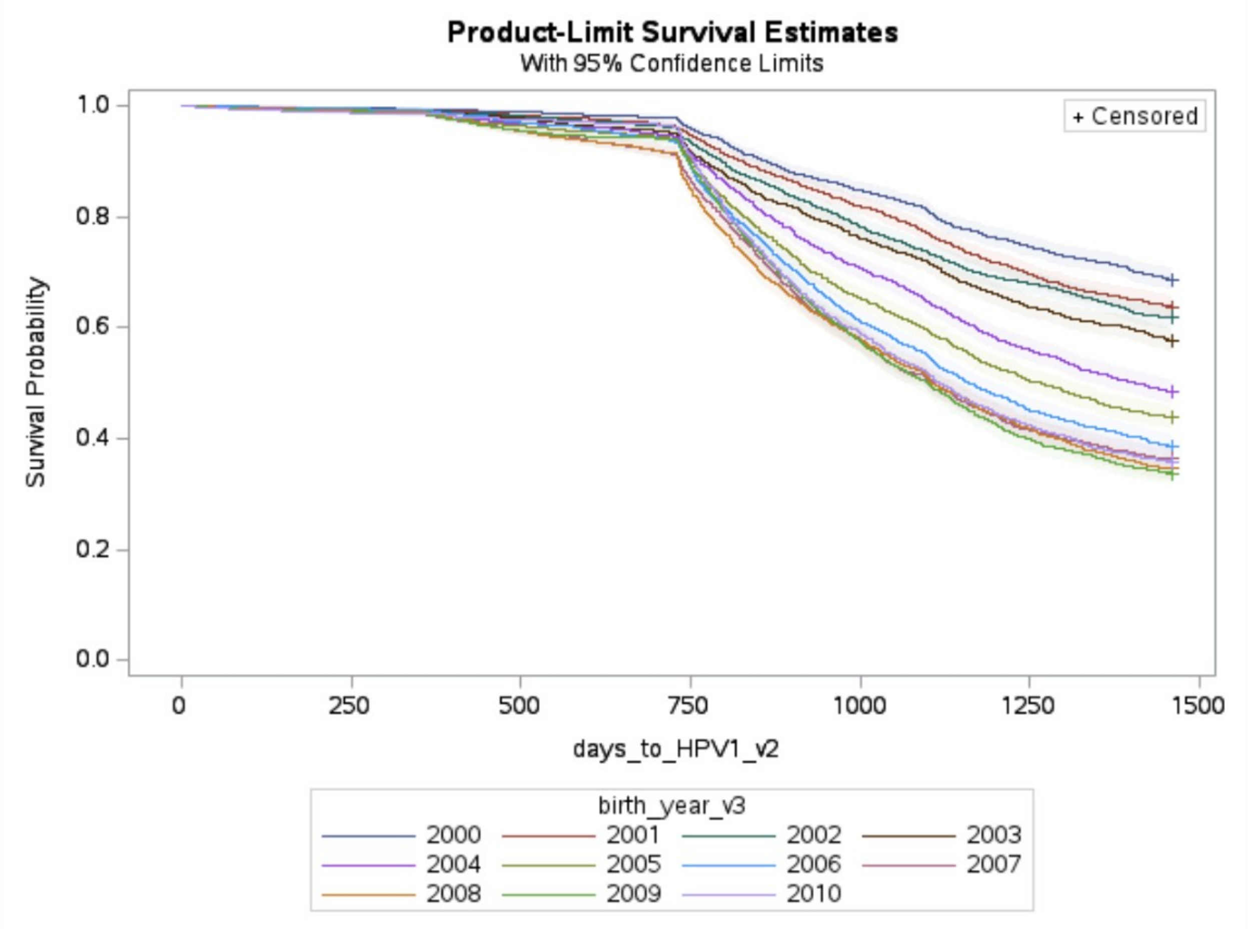

Appendix E: Kaplan-Meier curves – Tdap Vaccine Initiation (ages 11-12)

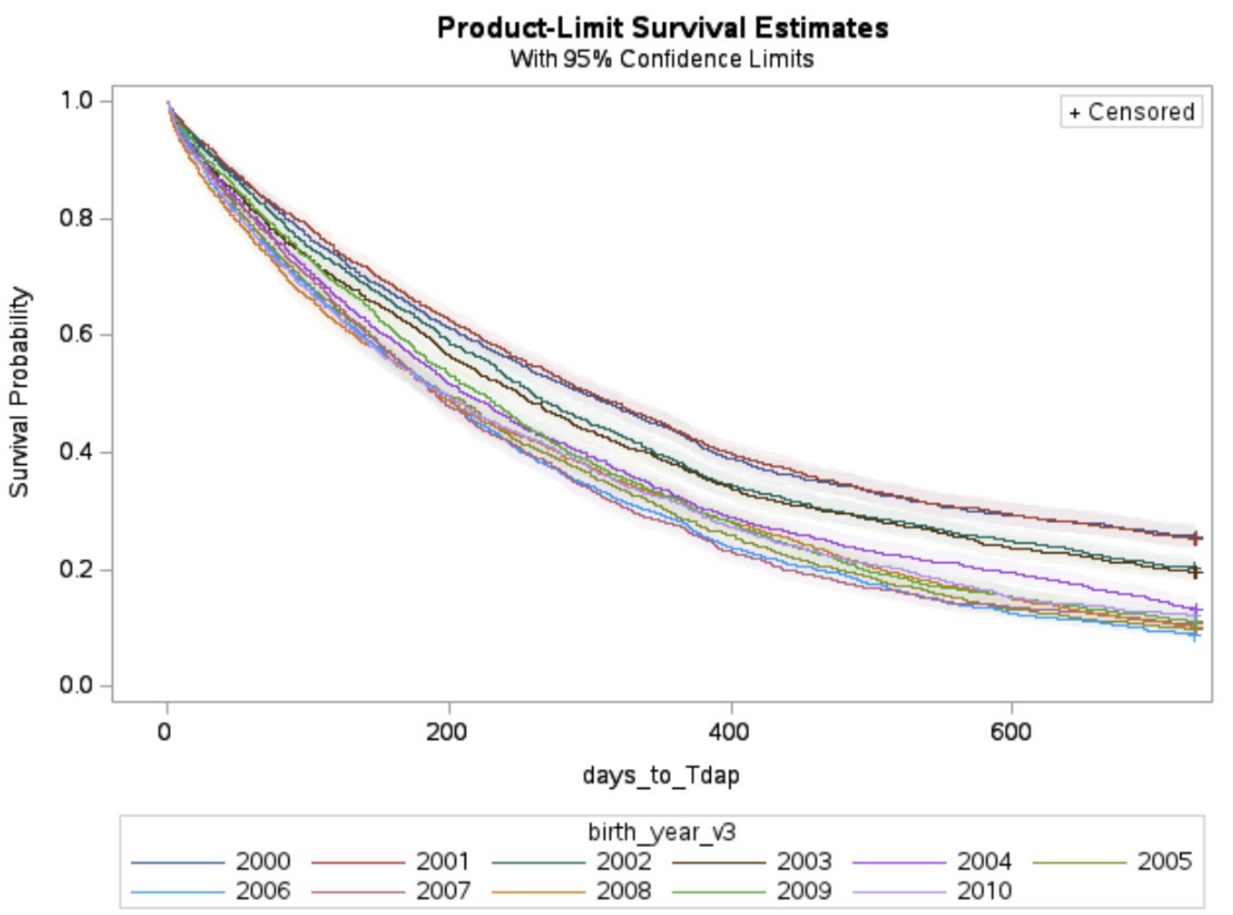

Appendix F: Kaplan-Meier curves – MenACWY Vaccine Initiation (ages 11-12)

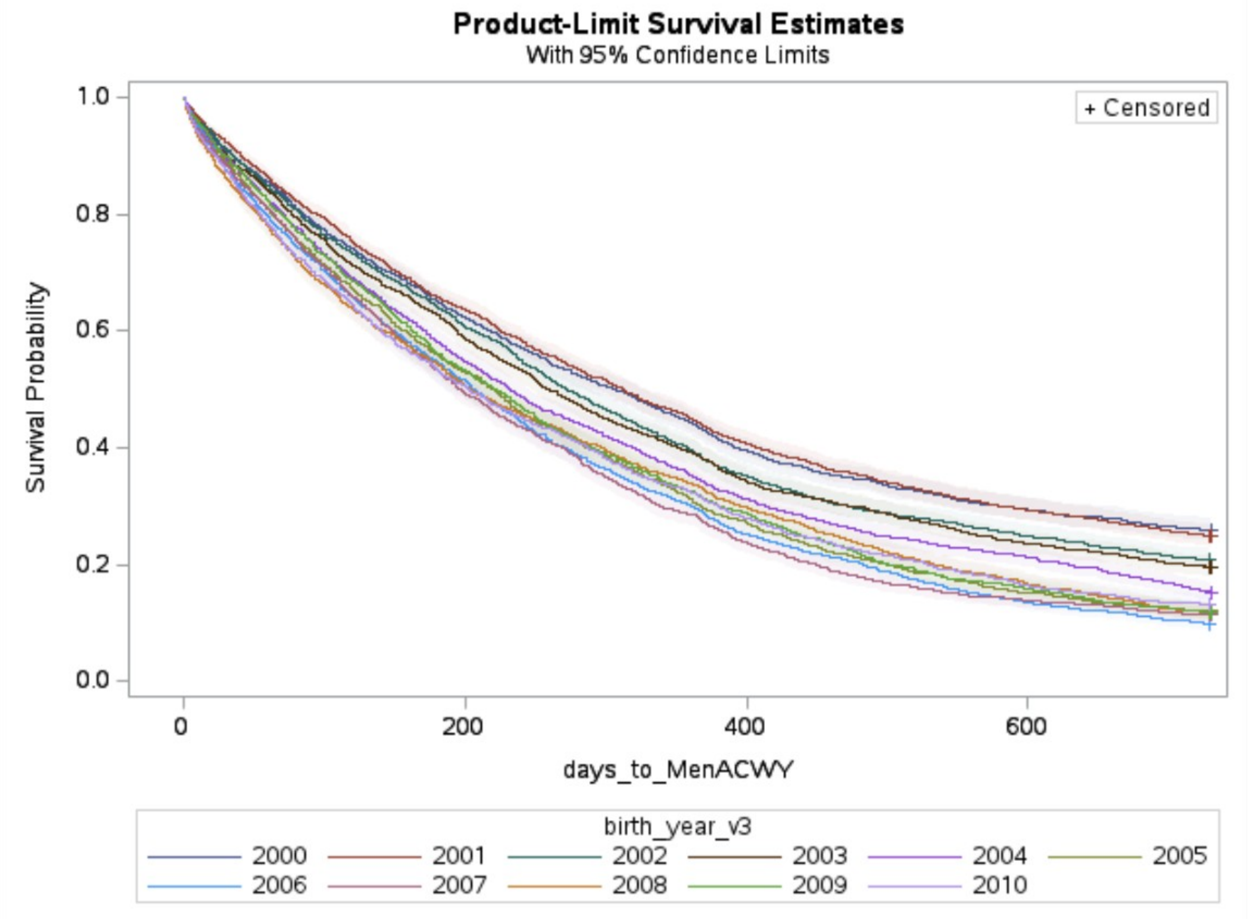

Appendix G: Kaplan-Meier curves, log-negative-log curves, and log-rank test p-values by sex, race, ethnicity, and insurance – HPV Vaccine Initiation (ages 9-12)

i. Sex

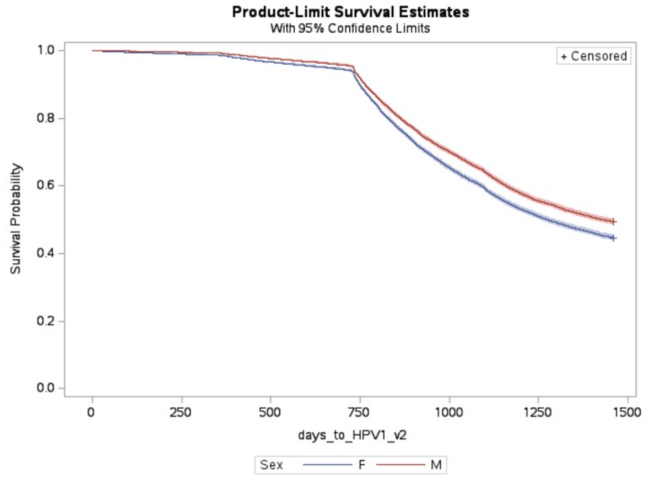

a.

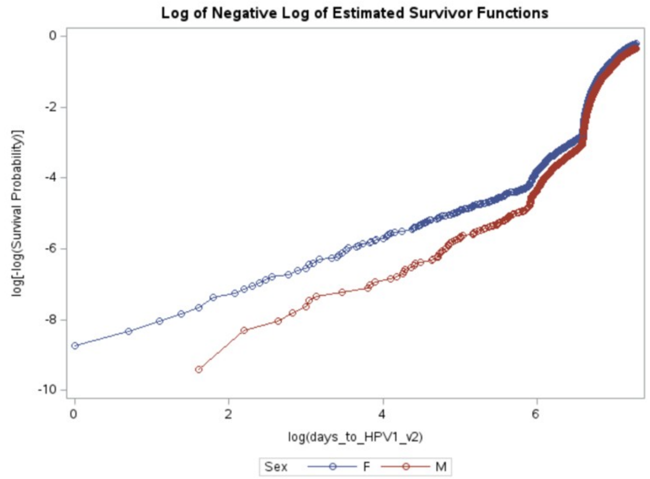

b.

c. Log-rank test p-value: p<0.0001

ii. Race

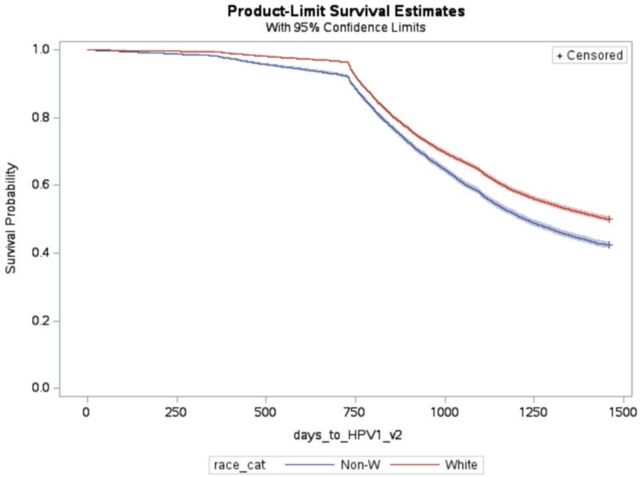

a.

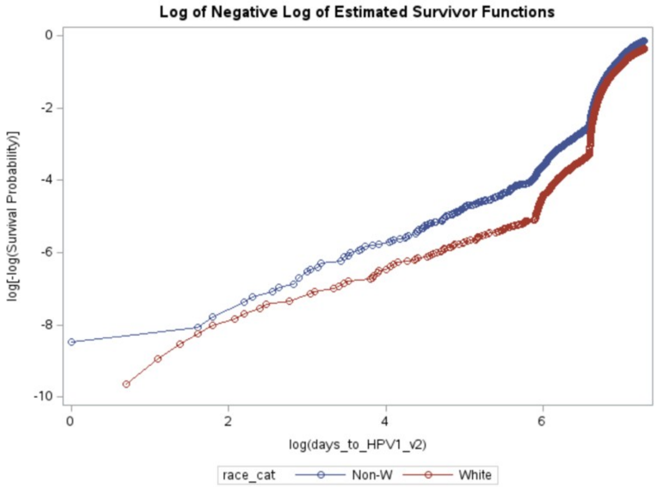

b.

c. Log-rank test p-value: p<0.0001

iii. Ethnicity

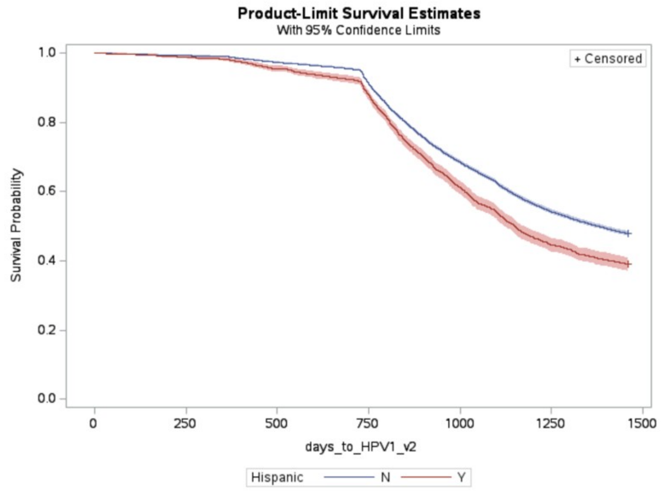

a.

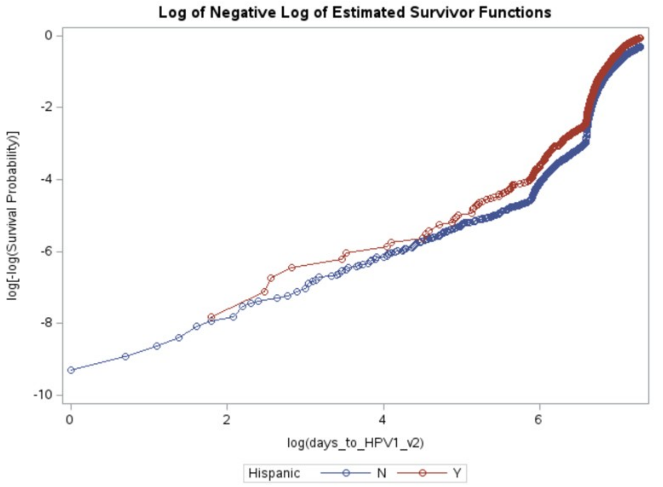

b.

c. Log-rank test p-value: $p<0.0001$

iv. Insurance

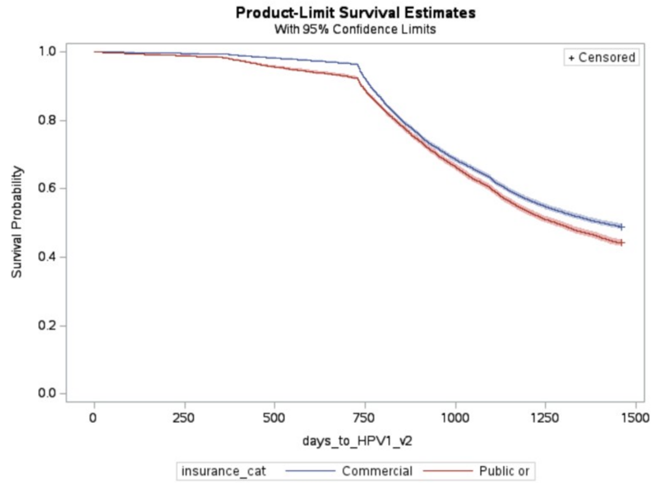

a.

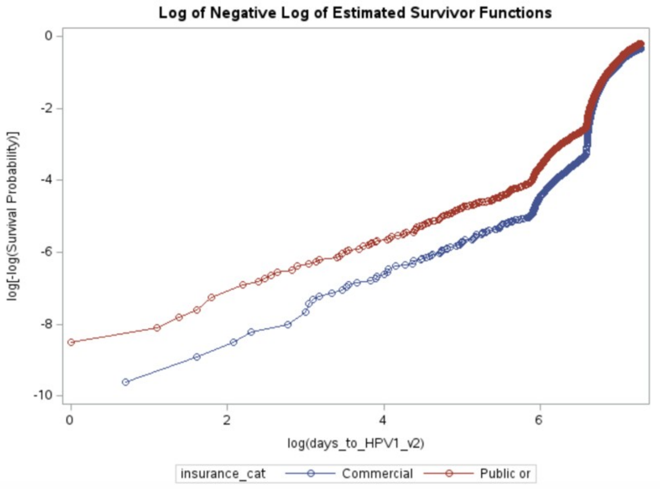

b.

c. Log-rank test p-value: p<0.0001

Appendix H: Kaplan-Meier curves, log-negative-log curves, and log-rank test p-values by sex, race, ethnicity, and insurance – Tdap Vaccine Initiation (ages 11-12)

i. Sex

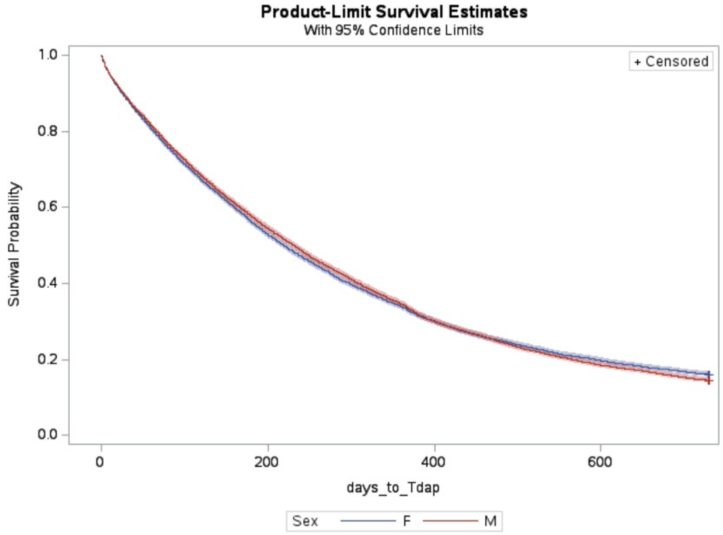

a.

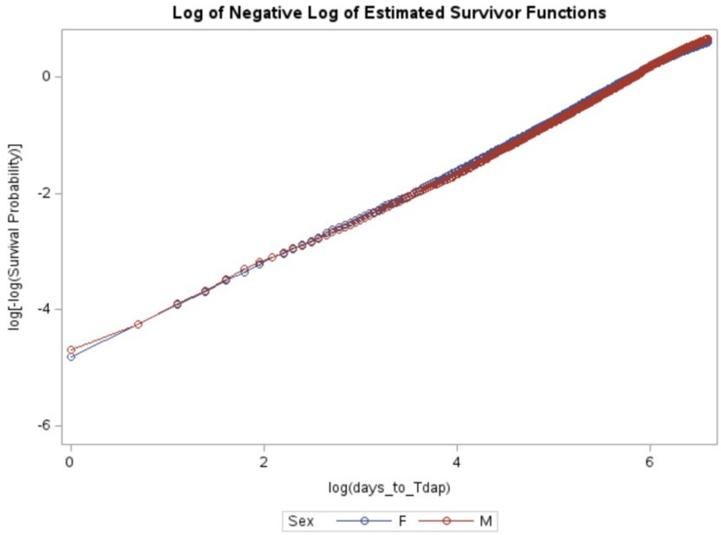

b.

c. Log-rank test p-value: 0 = 0.4542

ii. Race

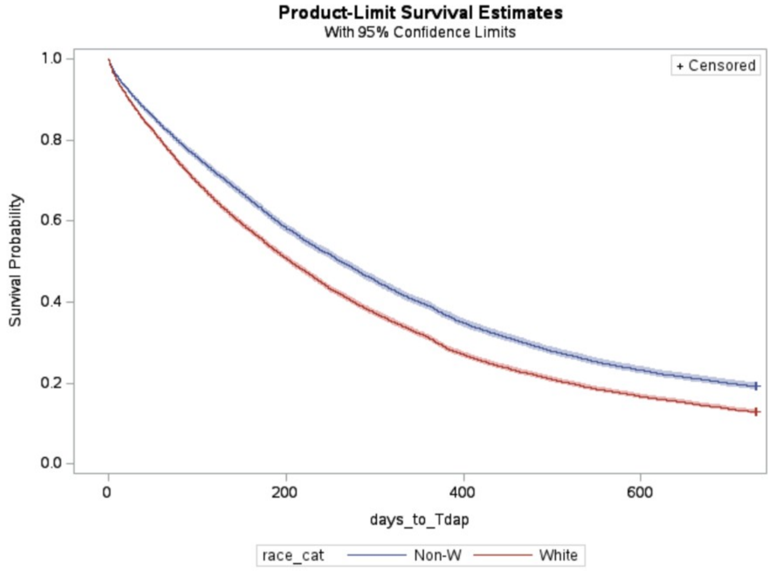

a.

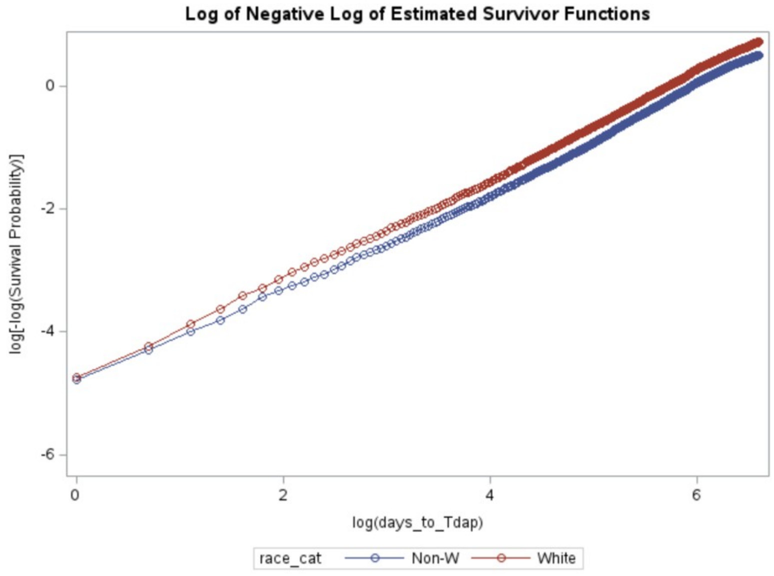

b.

c. Log-rank test p-value: p<0.0001

iii. Ethnicity

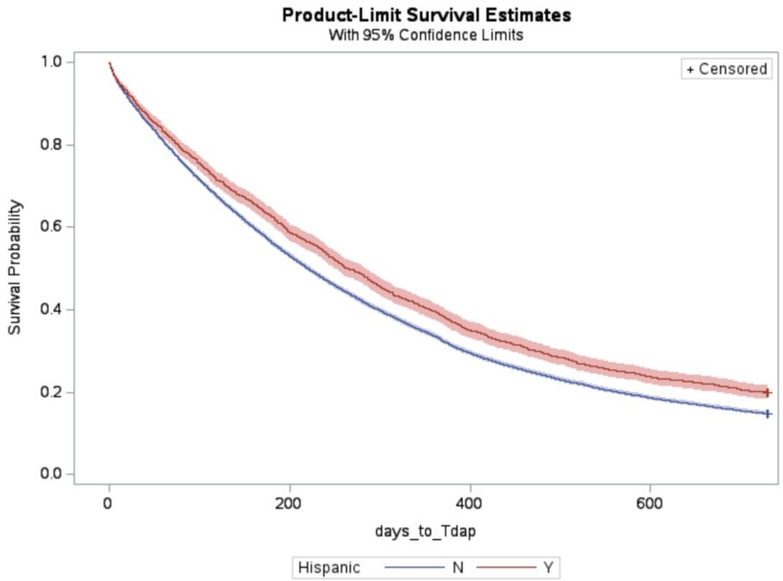

a.

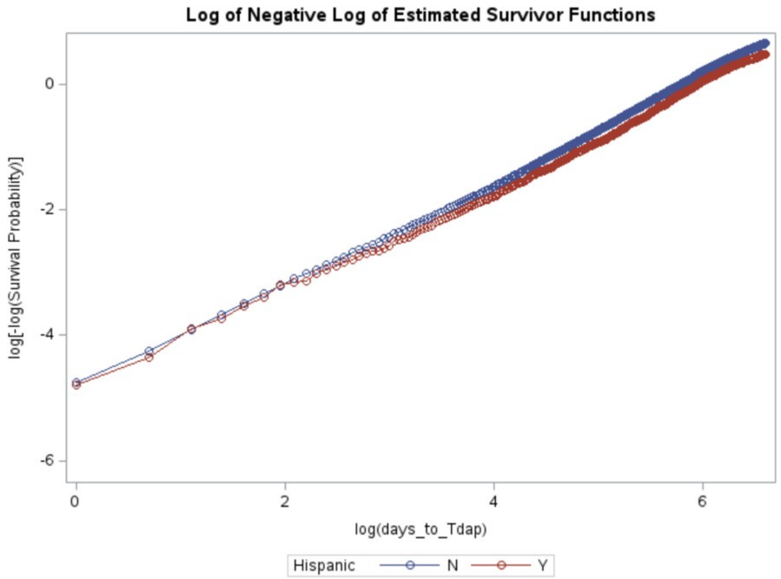

b.

c. Log-rank test p-value: $p<0.0001$

iv. Insurance

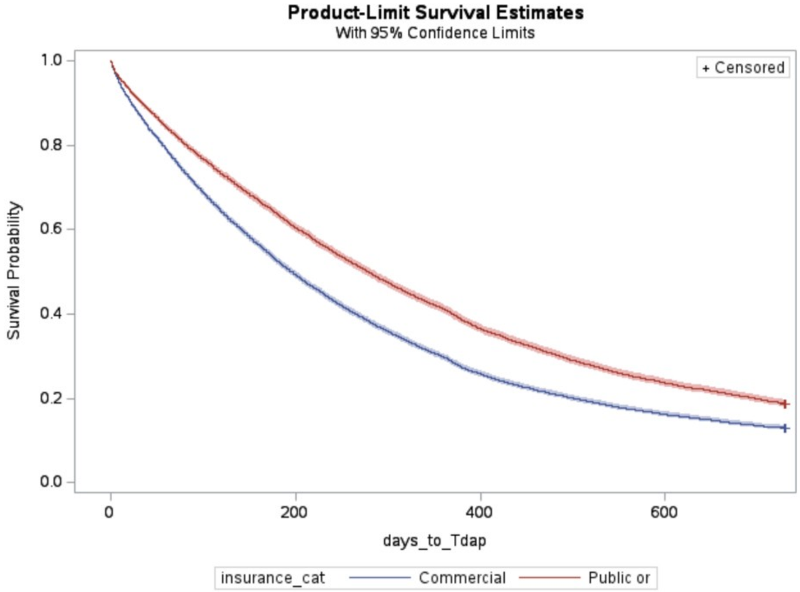

a.

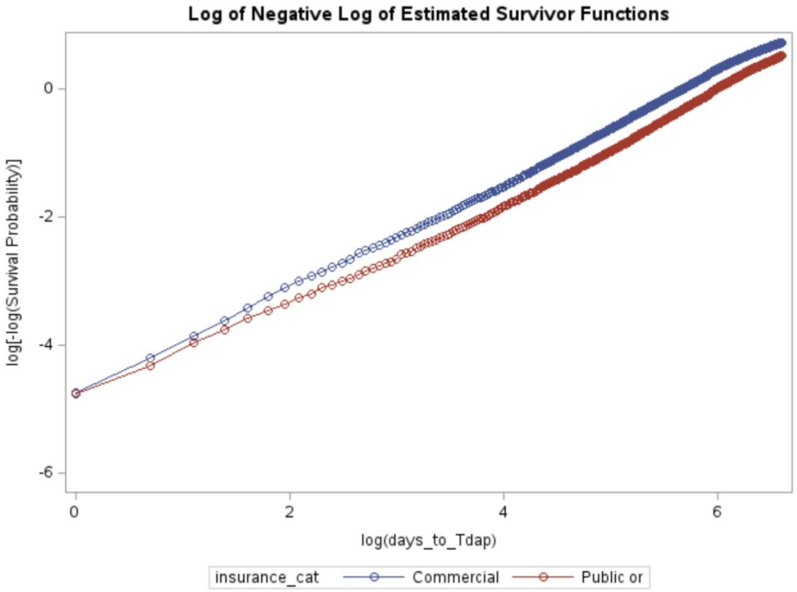

b.

c. Log-rank test p-value: p<0.0001

Appendix I: Kaplan-Meier curves, log-negative-log curves, and log-rank test p-values by sex, race, ethnicity, and insurance – MenACWY Vaccine Initiation (ages 11-12)

i. Sex

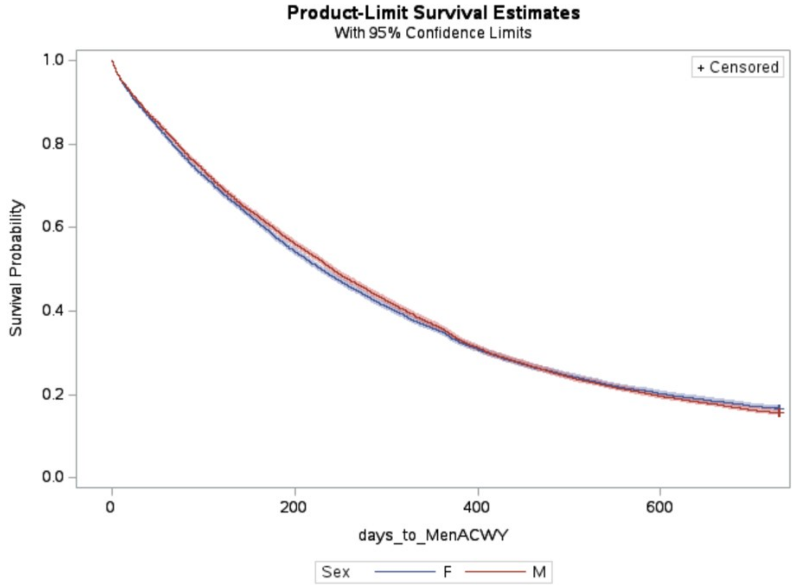

a.

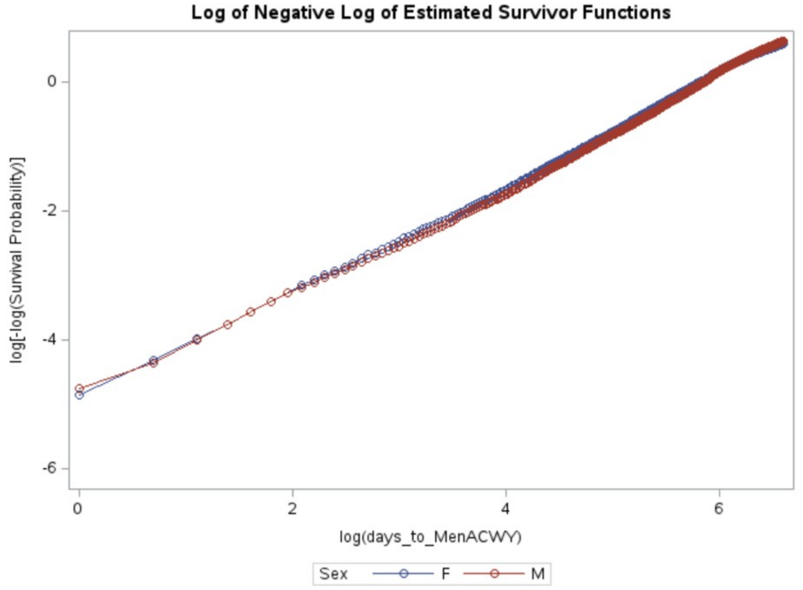

b.

c. Log-rank test p-value: p = 0.9770

ii. Race

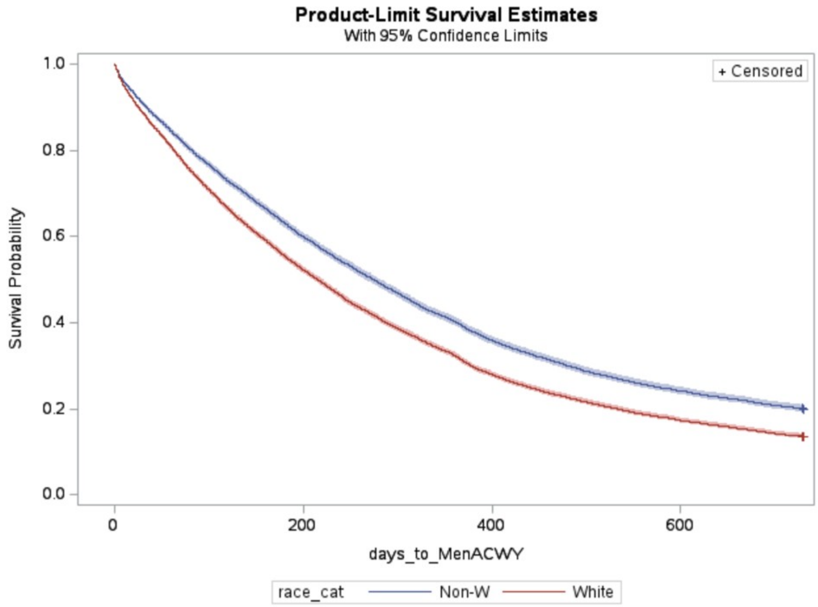

a.

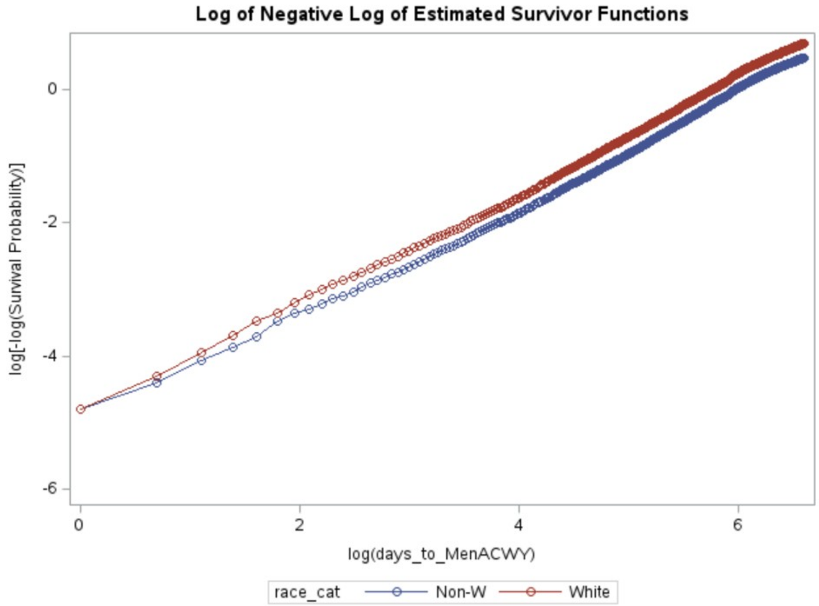

b.

c. Log-rank test p-value: p<0.0001

iii. Ethnicity

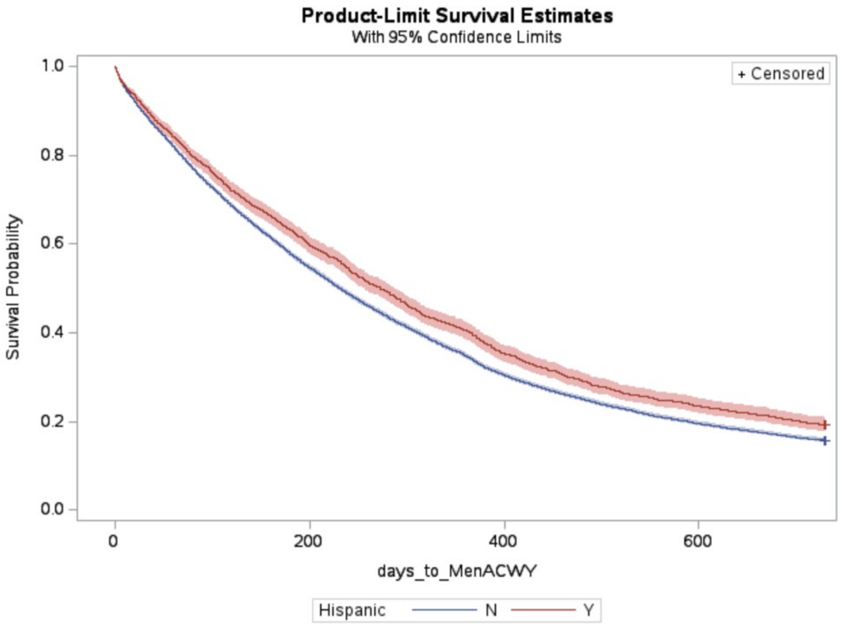

a.

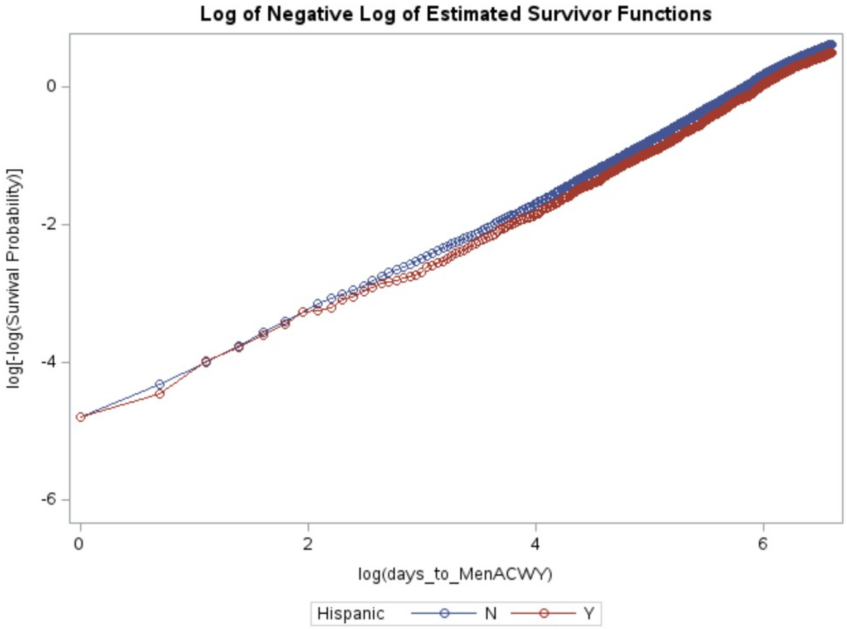

b.

c. Log-rank test p-value: $p<0.0001$

iv. Insurance

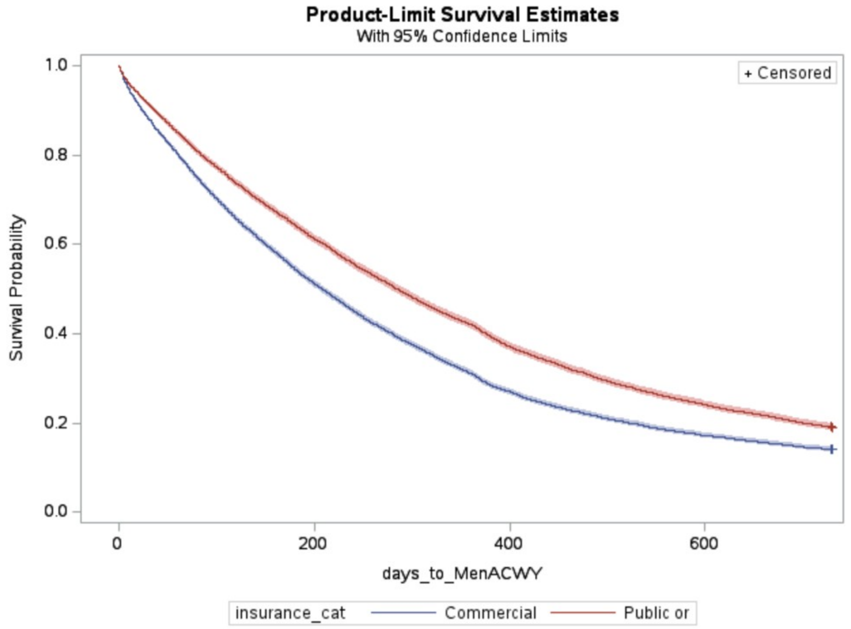

a.

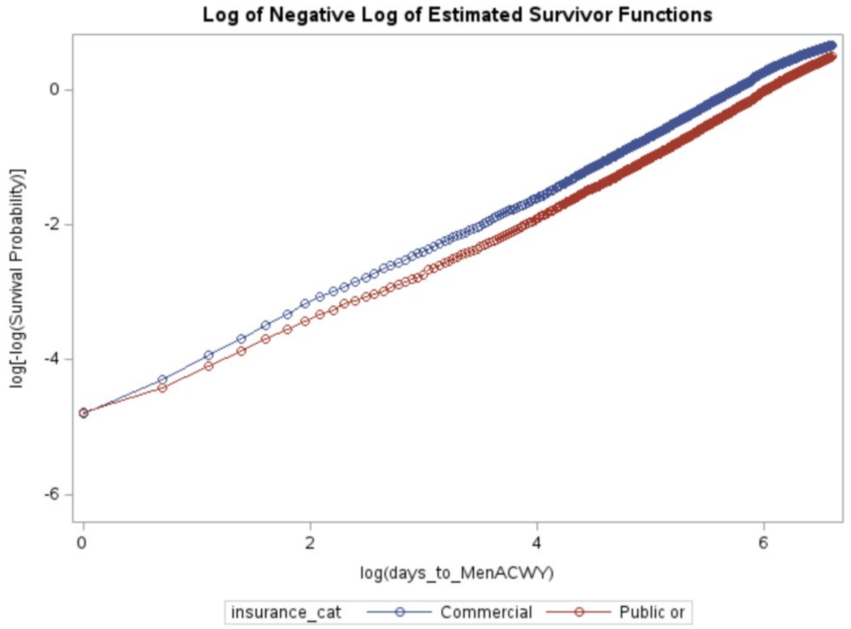

b.

c. Log-rank test p-value: $p<0.0001$
